# Evaluating water-yield property of karst aquifer based on the AHP and CV

**DOI:** 10.1038/s41598-022-07244-x

**Published:** 2022-02-28

**Authors:** Shuai Yu, Hanghang Ding, Yifan Zeng

**Affiliations:** 1 College of Geoscience and Surveying Engineering, China University of Mining and Technology (Beijing), Beijing, 100083 China; 2 National Engineering Research Center of Coal Mine Water Hazard Controlling, Beijing, 100083 China; 3 Experimental School Affiliated to Chinese Academy of Sciences, Beijing, 100020 China

**Keywords:** Environmental sciences, Hydrology

## Abstract

In order to ensure the safety of mine production, it is of great practical significance to make a reasonable evaluation of the water-yield property (WYP) of a karst aquifer. In this paper, we selected fault-lines distribution, fault-scale index, aquifer thickness, water pressure, consumption of rinsing liquid, and hydraulic conductivity as the evaluation indexes to analyze the WYP of a karst aquifer. Meanwhile, the analytic hierarchy process (AHP) is used to calculate the subjective weight of indexes, and the coefficient of variation (CV) is used to calculate the objective weight of indexes. Combined with GIS, a multi-factor composite superposition is carried out to evaluate the WYP of a karst aquifer. The reliability of the research results is verified by the specific yield. Besides, for improving the reliability of evaluation results, the chemical composition of karst water was discussed. The results show that the selection of indexes is reasonable and the AHP–CV method is effective to evaluate the WYP of a karst aquifer. Therefore, on the premise of reasonable index selection, the evaluation models of AHP and CV can be used to evaluate the WYP of a karst aquifer and provide reference for coal mine water control measures.

## Introduction

China is rich in coal resources and has a vast geographical distribution, but the hydrogeological conditions of coal mines are complex^1,2^. The influence of coal mining on groundwater resources is generally significant, which brings a serious threat to the safety of coal mining^3,4^^.^ In the process of mine construction and mining, the aquifer of roof and floor will be disturbed, therefore it is normal and inevitable that water-inrush occurs in the mine^5^. In general, the mining water-inrush depends on two aspects: one is the capacity building of mine drainage system; the other is the cognitive degree of mine water-filling conditions^6^. The primary problem of the water-filling condition is a source of water-filling, especially the distribution law of groundwater as a water-filling source and the WYP, which is an important factor causing frequent mine water disasters. If the water-filling aquifer exposed by mining is a weak water-yield area, water-inrush will not be induced generally. On the contrary, if the water-filling aquifer exposed by mining is a strong water-yield area, the instantaneous huge water-inrush disaster is likely to happen or even cause well flooding accidents. At present, the main evaluation methods of WYP can be divided into two ways^7–10^. One is to analyze the water-yield of aquifer using hydrogeological characteristics according to the geological data revealed by the borehole. Wu et al.^11^ put forward the water-yield index method of multi-information fusion based on GIS to analyze the aquifer. Yin et al.^12^ evaluated the water-yield of sandstone aquifer by using the analytic hierarchy process and trapezoidal fuzzy number. The other is to divide the aquifer into different zones by means of geophysical prospecting. Shi et al.^13^ used three-dimensional high-density electrical detection technology to realize the aquifer water-yield of the working face floor. Qiu et al.^14^ used the method of combination of gray correlation method and geophysical exploration to evaluate the water-yield of the Ordovician limestone karst aquifer.

With the deepening of coal mining depth, much more attention has been paid to karst water-inrush in the coal seam floor. How to address this problem has an important theoretical guiding significance and practical value for mine safety production. The comprehensive analysis method of the multi-index needs to reasonably select the indexes and determine the comprehensive importance of each index in the final evaluation results^15–17^. The object of evaluation is to determine the aquifer water-yield zoning. However, in determining the weight of indexes, human subjectivity makes the weight of some indexes prominent in the evaluation process, which affects the objectivity of evaluation, especially for many quantitative indexes^18^. In addition, two methods are usually used to verify the rationality of the evaluation results of water-yield^19,20^. There are specific yield and geophysical exploration. For the evaluation of large-scale WYP, it is obvious that geophysical exploration has limitations in scope.

Due to the high heterogeneity, anisotropy, and discontinuity of the permeability of the karst fissure water-filling aquifer in carbonate rock, the water-yield of the aquifer is extremely uneven^21^. With the characteristics of discontinuous distribution, and even in an aquifer, a unified surface of the groundwater head cannot be formed^22^. Therefore, it is necessary to further consider whether it is reasonable to use only a specific yield to verify the water-yield of a karst aquifer. Importantly, for Karst confined aquifer, reasonable evaluation indexes play an essential role to build an evaluating model.

Ordovician limestone belongs to floor confined aquifer, and there are few relevant methods to predict and evaluate the WYP of this aquifer. At present, we can find some researches using AHP method to study the WYP of roof sandstone or limestone aquifer, but the evaluation of WYP of Ordovician limestone aquifer is still in the form of geophysical exploration. Therefore, the workload in the early stage of mining is large and the buried depth leads to poor accuracy. If the geophysical exploration results can be compared with the evaluation model results, the prediction accuracy can be greatly improved. In order to study the water-yield of a karst aquifer, this paper adopts the method of combination of analytic hierarchy process (AHP) and coefficient of variation (CV) to make a comprehensive analysis using GIS composite Superposition Technology^23–28^. According to the way of weight formation, the weight can be divided into subjective weight and objective weight. Objective weight is the weight obtained by changing the expression form of statistical data and the synthesis way of statistical indexes. Otherwise, subjective weight is to determine the importance of each indicator according to the research purpose and the status of evaluation indexes. In this study, first, we used the AHP to calculate the subjective weight, and the CV to calculate the objective weight. Second, fault-lines distribution, fault-scale index, aquifer thickness, water pressure, consumption of rinsing liquid, and hydraulic conductivity are selected as the evaluation index, which can not only reflect the basic hydrological characteristics of the karst aquifer but reflect the influence of different karst development degree of the karst aquifer. Third, based on the analysis of specific yield and water chemical composition, the validity and rationality of water-yield index integrating AHP and CV are discussed from the perspectives of supply, runoff, and discharge. Last, through the proposed evaluation model of water-yield, we can make a reasonable evaluation of the water-yield law of karst aquifer, which has an extremely important theoretical guiding significance and practical value for mine safety production.

## Material and methods

### Study area

#### Location

**Figure 1:**
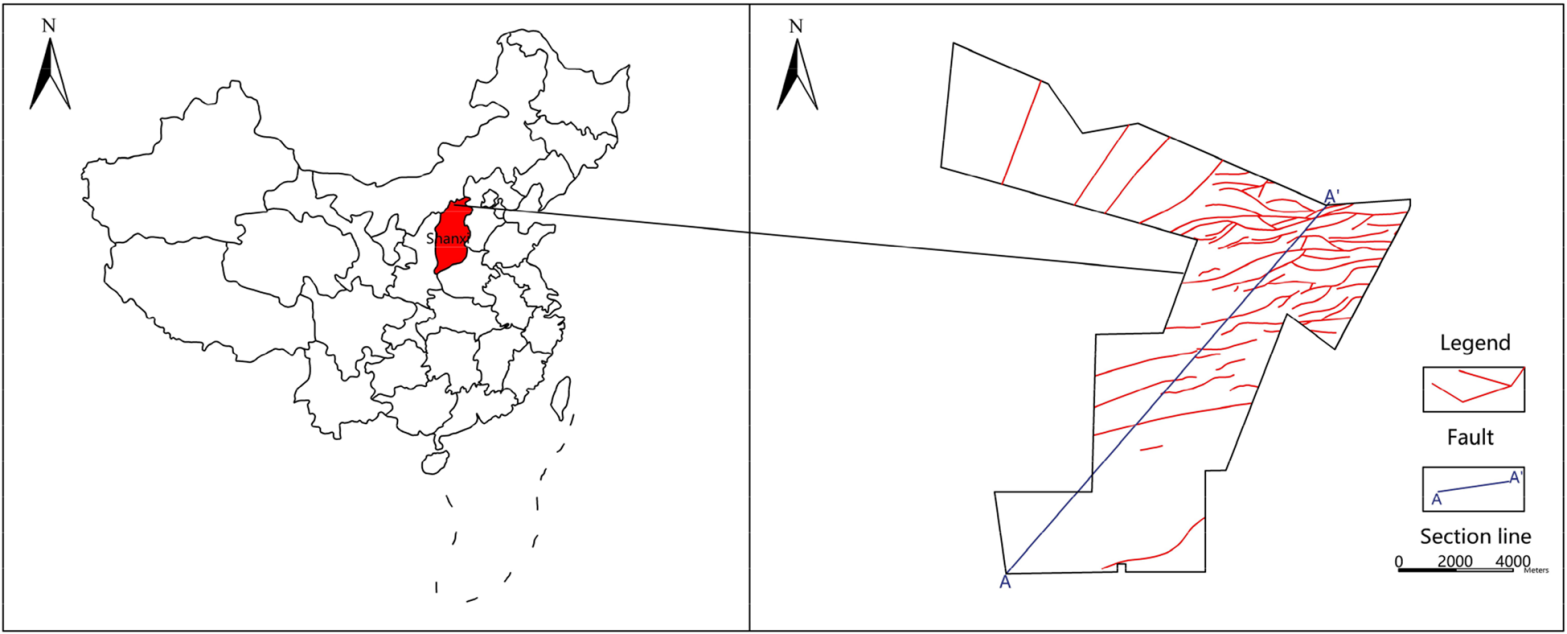
Location and structural geology map. Images are created using the Coreldraw, http://www.coreldraw.com/en/?link=wm.

Beixinyao coalfield is 11.091 km wide from east to west, 12.595 km long from south to north, with an area of 53.2980 km^2^ and a production capacity of 4.00 Mt/a. It is located at the junction of Yang Fangkou Town, Ningwu county and Bei Xinyao Town, Shuozhou city in China. Its geographical coordinates are 112°15′58″ to 112°23′39″ E longitude and 39°02′01″ to 39°08′50″ N latitude (Fig. 1). This area belongs to a continental climate, which is characterized by low annual average temperature, drought and less rain, windy sand in winter and spring, rainfall concentrated in summer, evaporation is greater than precipitation.

#### Geology and hydrogeology

**Figure 2:**
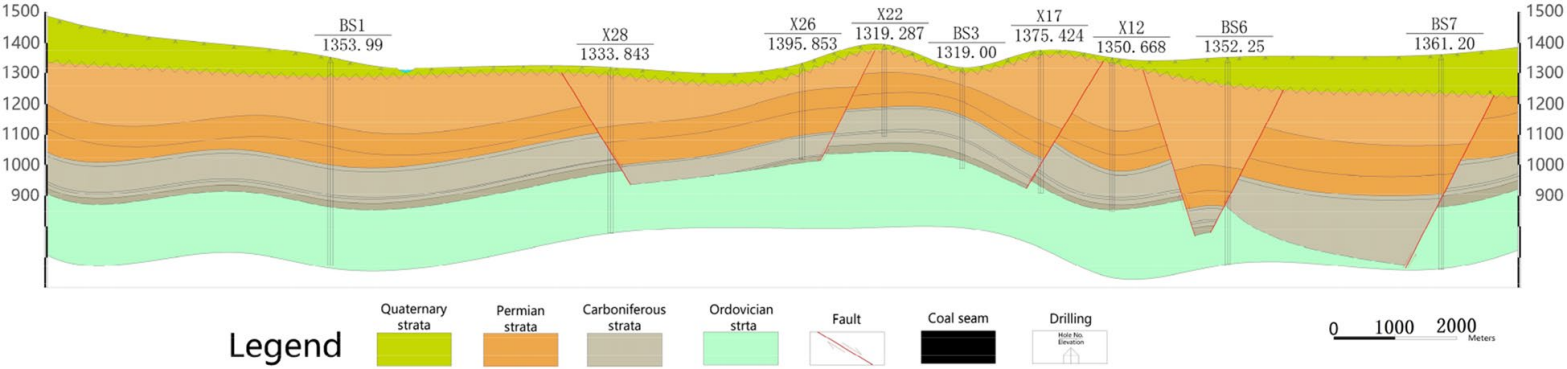
Geological cross-section (A–A′) of the study area.

Most of the coalfield is covered by loess, only a few upper Permian Shihezi Formation are exposed in some gullies, and the upper and lower Ordovician Majiagou formation is exposed in the northwest edge and periphery of the minefield. According to the surface and borehole exposure, the sedimentary strata in the coalfield are successively from the old to the new: the Majiagou Formation of the Middle Ordovician system, the Benxi Formation and the Taiyuan Formation of the Carboniferous system, the Shanxi Formation and the Shihezi Formation of the Permian system, the Quaternary system. Affected by regional structure, faults in the minefield are relatively developed (Fig. 1). Figure 2 shows the typical geological cross-section of the study area.

Six aquifers are deposited in the mine area, from new to old, there are pore aquifer of Quaternary loose layer, sandstone fracture aquifer of Shihezi Formation and sandstone fracture aquifer of Shiqianfeng Formation, sandstone fracture aquifer of Shanxi Formation, sandstone fracture aquifer of Taiyuan Formation, limestone fracture aquifer of Majiagou Formation. They can be divided into three groups according to the type of aquifer media. The aquifer studied in this paper is the third aquifer that is a limestone fractured aquifer.

### Data

#### Fault-lines distribution

**Figure 3:**
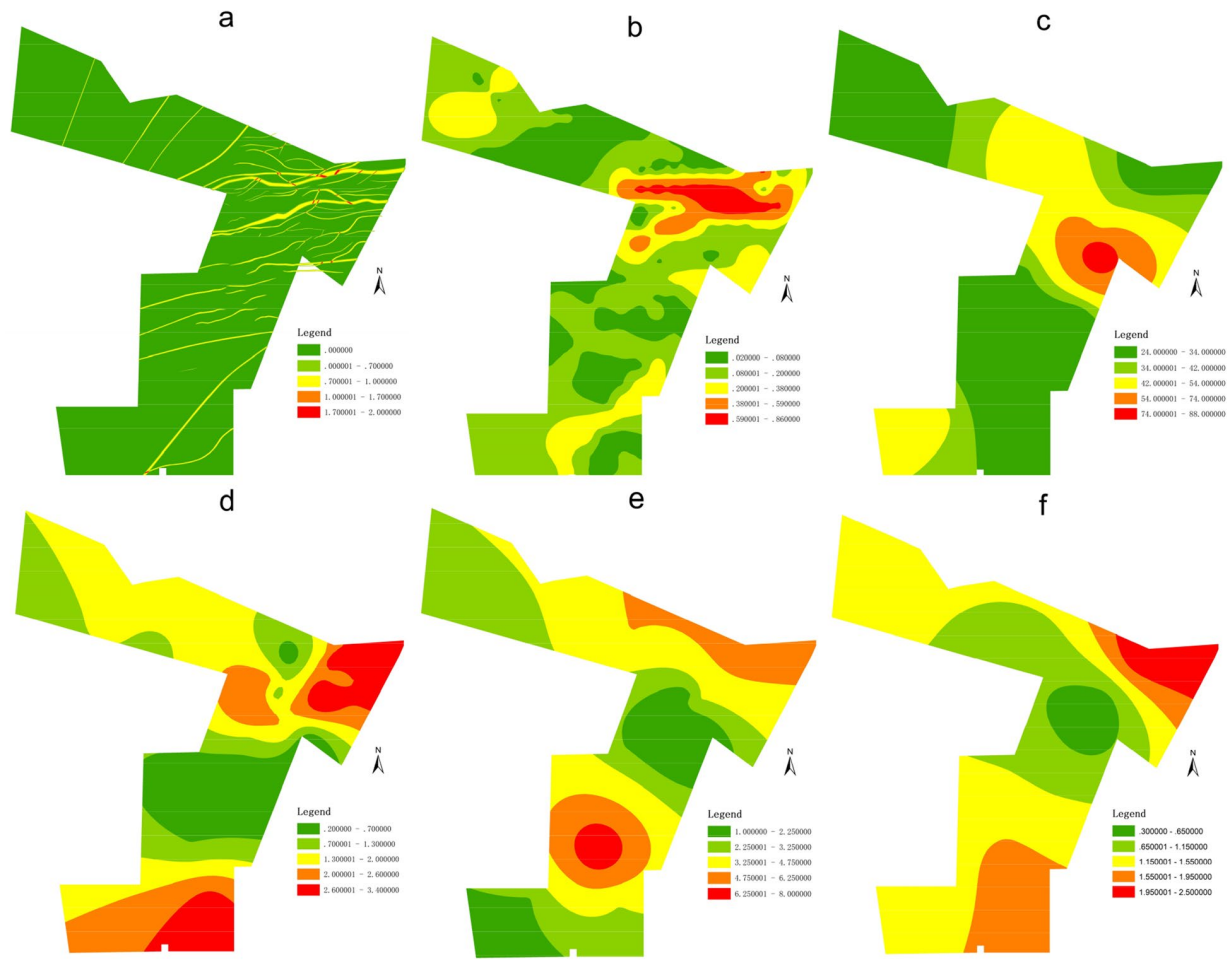
Influence factors of WYP: (**a**) Fault-lines distribution, (**b**) Fault -scale index, (**c**) Aquifer thickness (m), (**d**) Water pressure (Mpa), (**e**) Consumption of rinsing liquid (m^3^/h), (**f**) Hydraulic conductivity (m/d).

Faults will destroy the integrity of the aquifer and produce many fractures. Also, the rock near the fault zone is relatively broken under the influence of structural stress, which provides a good channel for surface water and groundwater. Moreover, the karst development of limestone in the fault developed area is more intense. On the one hand, many fractures are formed in the broken rocks, which provide water transport channels; on the other hand, the surface area of the rocks contacting with water is increased, which provides necessary conditions for the development of karst. Therefore, the aquifer in the structural development area has a good water-yield property. In other words, the fault-lines distribution (FLD) is an essential factor (Fig. 3a). The fractures in the fracture zone of the normal fault interrupt layer are more developed than the fault influence zone. According to previous study, the value of 1 is assigned to the fracture zone and 0.7 to the influence zone during quantification, which is opposite in reverse fault. When quantifying the intersection of two faults, if the distribution area of the fracture zone of the two faults is A; The distribution area of the influence zone is B. for example, after the intersection and superposition of two normal faults, the generated three types of areas AA, AB and BB correspond to the intersection quantization values 2, 1.7 and 1.4 respectively according to the quantification rules of fault distribution^11^.

#### Fault-scale index

Fault-scale index (FSI) reflects the overall scale and development degree of the fault, and the fracture is often developed in the large-scale fault development area (Fig. 3b). The larger the fault-scale index is, the larger the fault scale is, the better the development degree is. Because the development degree of karst is controlled by the development of fault to some extent, the scale index of fault can be used to express the development degree of karst. Therefore, FSI can be used to reflect the water-yield of a karst aquifer. The FSI is the sum of the product of the fall and length of all faults in a unit area. The expression is as follows:

1 $$FSI = \frac{{\mathop \sum \nolimits_{i}^{n} L_{i} H_{i} }}{S}$$

*H*_i_ is the fall of the fault (m); *L*_i_ is the strike length of the fault within the unit area (m); *n* is the number of faults falling in the unit; *S* is the unit area (500 × 500 m^2^).

#### Aquifer thickness

According to Darcy’s law, the water-yield of an aquifer is directly proportional to the thickness of the aquifer, that is, the greater the thickness of the aquifer, the stronger the water-yield of the aquifer (Fig. 3c). Because the karst aquifer of Middle Ordovician limestone is deeply buried and thick. The water-yield of karst aquifer is affected by karst development and has vertical distribution characteristics. The deeper the aquifer is buried, the better the integrity of limestone is, and karst is even not developed. Therefore, it is unreasonable to use the fully exposed Ordovician limestone thickness as the aquifer thickness. If the whole Ordovician limestone aquifer is not fully exposed in the mining area, on the one hand, there is no practical significance; on the other hand, it will cause waste of drilling resources and excessive economic waste. In view of the hydrogeological borehole data of the mining area, the aquifer thickness exposed by the borehole is counted as an important factor.

#### Water pressure

Water pressure (WP) of the confined aquifer can also be used as an important index to reflect the water-yield of the aquifer (Fig. 3d). The larger the WP is, the greater the water content of the aquifer is. For confined aquifer, the WP is relatively stable without artificial drainage or natural damage. However, due to the heterogeneity of karst aquifer, the WP at different locations is also different, which reflects the heterogeneity of aquifer water-yield. Therefore, it is reasonable to use aquifer water pressure as a water-yield factor.

#### Consumption of rinsing liquid

Drilling rinsing liquid plays an important role in drilling engineering, except for cleaning and lubrication. The change of rinsing liquid consumption can reflect the lithology and water permeability of the rock stratum (Fig. 3e). Generally, in the process of geological drilling, it is necessary to observe the consumption of rinsing liquid at any time. If the nature and consumption of rinsing liquid have changed, it indicates that the permeability and leakage of the formation have changed, and a new aquifer (zone) may have been exposed. The consumption of rinsing liquid in a certain rock section also indicates the hydraulic conductivity and water inflow of the rock section. Therefore, it is of great significance to take the consumption of rinsing liquid as one of the multiple geoscience information reflecting the water-yield property of aquifer.

#### Hydraulic conductivity

Hydraulic conductivity is an important hydrogeological parameter, which is a quantitative index to characterize the permeability of rock (Fig. 3f). The hydraulic conductivity depends not only on the properties of rock but also on the physical properties of the permeable liquid. However, the permeability of groundwater depends on the physical properties of rock when the physical properties of groundwater are unchanged. The hydraulic conductivity reflects the ability of the rock to allow water to pass through. Therefore, the more broken the rock, the greater the hydraulic conductivity and the stronger the water permeability. It can also improve the rate of hydraulic exchange.

## Methods

### Subjective weight of AHP

AHP divides the interrelated elements into several levels according to the membership. The experienced experts are invited to give quantitative indexes for the relative importance of each level and factor, and then use the mathematical method to synthesize the expert opinions and give the relative importance weight value of each level and factor as the basis of the comprehensive analysis^23^.

#### Building hierarchy model

**Figure 4:**
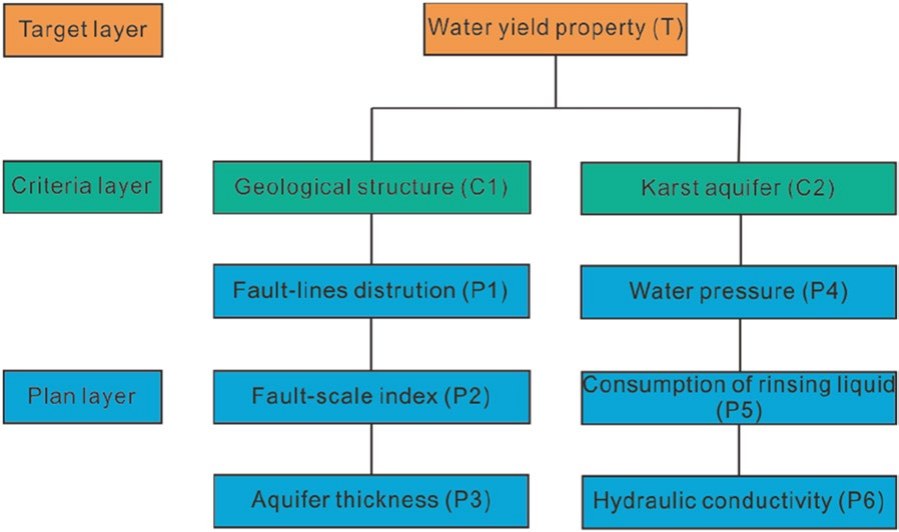
Hierarchical structure model of the study area.

AHP divides the factors to be considered in the system into several groups according to their attributes, each group as a layer, and the elements of the same level as the criteria play a dominant role in some elements of the next layer. At the same time, they are dominated by the elements of the previous layer. This dominant relationship from the top to the bottom constitutes a recursive hierarchy^24,28^. Therefore, according to the geological characteristics of the research area, the hierarchical structure model of the research objectives is as Fig. 4.

#### Constructing judgment matrix

When determining the weight of factors at all levels, AHP does not put all factors together for comparison, but compares them with each other. In order to show the importance of each element in the matrix quantitatively, the element *a* in the matrix can be used 1–9 scale method. All the comparison results can be expressed by the comparison matrix *A*:

2 $$A = \left[ {\begin{array}{*{20}c} {a_{11} } & \cdots & {a_{1j} } \\ \vdots & \ddots & \vdots \\ {a_{i1} } & \cdots & {a_{ij} } \\ \end{array} } \right],\quad a_{ij} = \frac{1}{{a_{ji} }}$$

The eigenvector corresponding to the maximum characteristic root of the judgment matrix is the weight. In this paper, the weight calculated by AHP is redefined as subjective weight (SW).

3 $$SW = \left( {w_{1} ,w_{2} , \ldots w_{n} } \right)^{T}$$

#### Matrix consistency test

Using the maximum eigenvalue as the weight vector of the influence degree of the compared factor on the upper level factor. The greater the degree of inconsistency, the greater the judgment error. The consistency indexes (CI) are defined as:

4 $$CI = \frac{\lambda - n}{{n - 1}}$$

In order to measure the size of CI, the random consistency index (RI) is introduced.

5 $$RI = \frac{{CI_{1} + CI_{2} + \cdots + CI_{n} }}{n}$$

Considering that the deviation of consistency may be caused by random reasons, when checking whether the judgment matrix has satisfactory consistency, it is also necessary to compare CI with random consistency index RI to obtain the test coefficient CR, which is as follows according to Eqs. (4) and (5).

6 $$R = \frac{CI}{{RI}}$$

Generally, if CR < 0.1, the judgment matrix is considered to pass the consistency test, otherwise it will not have satisfactory consistency.

### Objective weight of CV

Coefficient of variation (CV) is a statistic to measure the degree of variation of each observation value in the data^7,29^. When comparing the degree of variation of two or more data, if the unit of measurement is the same as the average, the standard deviation (SD) can be used directly for comparison. If the unit and the average are different, the SD cannot be used to compare the degree of variation, however, the ratio of the standard deviation and the mean (MN) can be used. The relative standard variation coefficient is the ratio of the variation index of a group of data to its average index, which is a relative variation index^26^.

7 $$V_{i} = \frac{{\sigma_{i} }}{{\overline{x}_{i} }}\quad \left( {{\text{i}} = {1},{ 2}, \, \ldots ,{\text{ n}}} \right)$$

where $V_{i}$ is the coefficient of variation of index *i*, $\sigma_{i}$ is the standard deviation of index *i*, and $\overline{x}_{i}$ is the average of index. Therefore, objective weight (OW) can be calculated by Eq. (8).

8 $$OW_{i} = \frac{{V_{i} }}{{\mathop \sum \nolimits_{i = 1}^{n} V_{i} }}$$

### Comprehensive weight

Comprehensive weight (CW) is a method to couple AHP and CV, and the calculation formula is as follows:

9 $$CW = kSW + \left( {1 - k} \right)OW$$

*k* is the preference coefficient. In this study, $k = 0.5$^30^.

### Data normalization

In order to eliminate the influence of the data of different dimensions of the main control factors on the evaluation results, it is necessary to normalize the data. The purpose of normalization is to make the data comparable, statistically significant and convenient for systematic analysis^6^.

10 $$A_{i} = \frac{{x_{i} - {\text{min}}\left( {x_{i} } \right)}}{{\max \left( {x_{i} } \right) - {\text{min}}\left( {x_{i} } \right)}}$$

where $A_{i}$ is the data after normalization; ${\text{max}}\left( {x_{i} } \right)$ and ${\text{min}}\left( {x_{i} } \right)$ are the minimum and maximum values of the quantification values of each main control factor respectively.

### Water-yield property index

Water-yield property index is to use multi-source information composite technology for composite superposition. The thematic map of each main control factor affecting the water-yield of an aquifer is represented by a circular domain, and there are three relationships among each circular domain: (1) the main control factors have an impact on the water-yield of an aquifer when they are completely overlapped at the same time, and the relationship of two main control factors is inclusion; (2) the main control factors have an impact on the water-yield of an aquifer when they are partially overlapped, and the relationship of two main control factors is intersect; (3) the main control factors have an impact on the water-yield of an aquifer respectively, and the relationship of two main control factors is mutually disjoint. The more circles overlap in a certain area, the more factors affect the aquifer water-yield.

The spatial information superposition function of GIS is used to synthesize the various factors reflecting water-yield into a quantitative index, which is called water-yield property index (WYPI). All factors are normalized by Eq. (10).

11 $$WYPI = \mathop \sum \limits_{i = 1}^{n} CW_{i} f_{i} \left( {x,y} \right)$$

where *CW*_i_ is the comprehensive weight of influencing factors; *f*_i_ (*x*, *y*) is the function of single factor influencing value; *x*, *y* is the geographical coordinate; *i* is the number of influencing factors.

## Results and discussions

### Results

#### Determining the CW

According to the calculation method of AHP, the judgment matrix of each level is shown in the Tables 1 and 2. the judgment matrix passed the consistency test by Eq. (6), CR < 0.1.

**Table 1:** Weight calculation of criteria layer.

T	C1	C2	Weight
C1	1	1/2	0.333
C2	2	1	0.667
$$\lambda_{max} = 2\quad CR = 0 < 0.1$$

**Table 2:** Weight calculation of plan layer.

C1	P1	P2	P3	Weight
P1	1	4	3	0.614
P2	1/4	1	1/3	0.117
P3	1/3	3	1	0.268
$$\lambda_{max} = 3.0735\quad CR = 0.0707 < 0.1$$				

C2	P4	P5	P6	Weight
P4	1	3	1/2	0.333
P5	1/3	1	1/3	0.140
P6	2	3	1	0.528
$$\lambda_{max} = 3.0536\quad CR = 0.0516 < 0.1$$				

Consequently, Combining the weight of OW, the results of CW are shown in the Table 3.

**Table 3:** Weight calculation of AHP–CV.

Weight	P1	P2	P3	P4	P5	P6
AHP	0.267	0.111	0.047	0.267	0.133	0.176
CV	0.152	0.096	0.302	0.160	0.153	0.136
AHP–CV	0.210	0.103	0.174	0.213	0.143	0.156

#### The model result of WYPI

**Figure 5:**
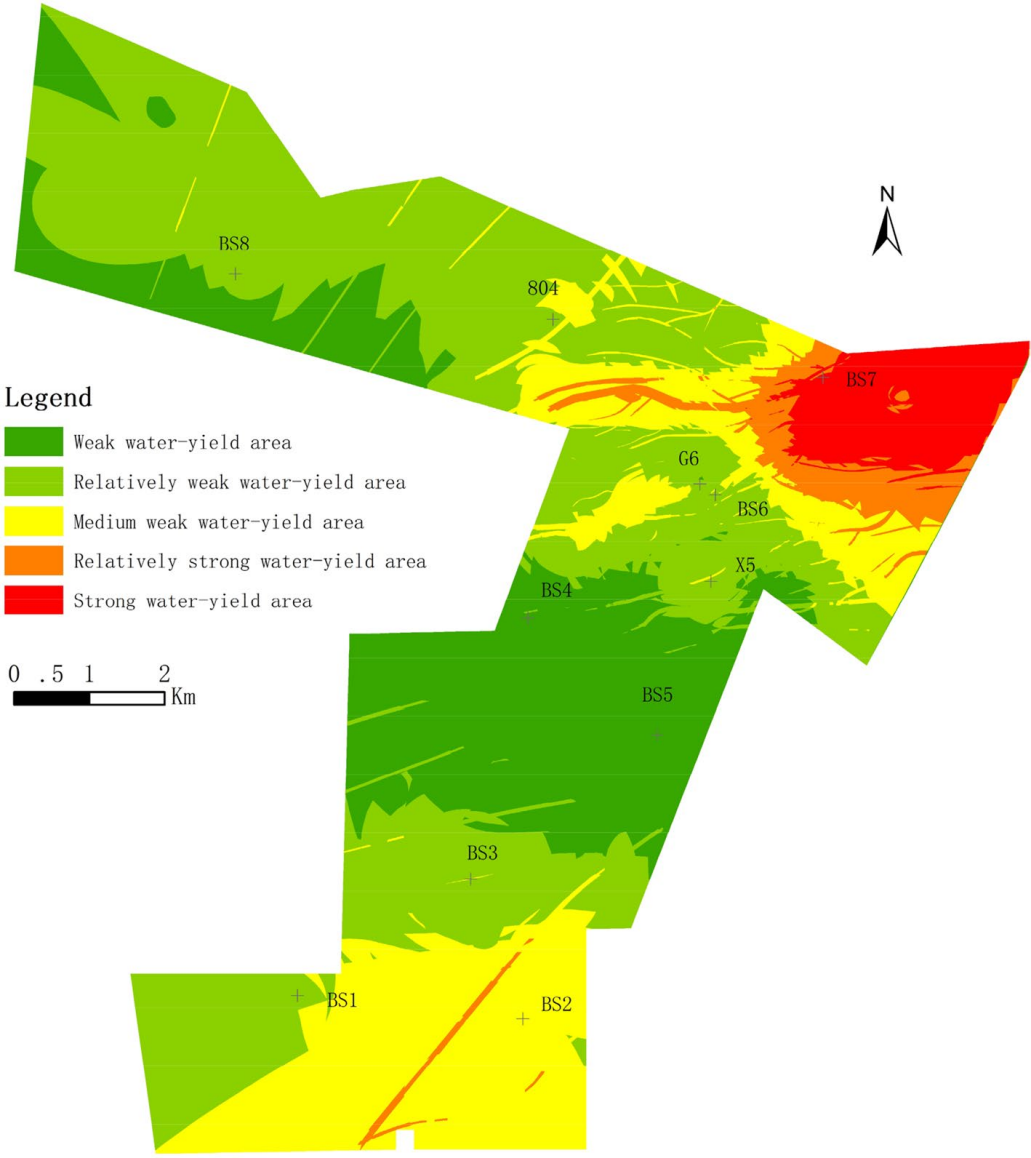
Water yield property zone map by AHP–CV.

The thematic maps of each information were superposed by combining the method of AHP–CV and using the spatial data analysis function of GIS, and the evaluation grade of each unit was calculated. According to the results, the WYP area was divided into five grades by Natural breakpoint classification method based on Jenks, which are strong water-yield area, relatively strong water-yield area, medium water-yield area, relatively weak water-yield area and weak water-yield area respectively. Natural breakpoint classification is a standard classification method in ArcGIS, which is based on the inherent natural grouping in the data, identifies the classification interval, and can group the similar values most appropriately, so as to minimize the intra class differences and maximize the inter class differences. Elements are divided into multiple classes according to the needs of users. For these classes, their boundaries will be set at locations with relatively large differences in data values^3^. The results showed that the strong water-yield area and the relatively strong water-yield area appeared in the northeast of the mining area. In addition to the distribution around the strong water-yield area in the northeast, the middle water-yield area is mainly concentrated in the south of the mining area, and the southern fault zone has a relatively strong water-yield area. At the same time, the northwest and the middle of the mining area are mainly relatively weak water-yield area and weak water-yield area (Fig. 5).

## Discussions

In this study, we have shown that a method was used to evaluate the WYP of a karst aquifer, which adopts a multi-factor composite superposition integrated AHP and CV. Fault-lines distribution, fault-scale index, aquifer thickness, water pressure, consumption of rinsing liquid, and hydraulic conductivity are selected as the evaluation indexes.

There are many methods used to determine the weight, each method has its advantages, and the ultimate purpose of the weight is to facilitate the interpretation of the influence ability of various factors under multi-factor conditions^11,19^. AHP is a widely used method for weight calculation, and its practicability has been well verified^18,28^. However, its shortcomings cannot be ignored, that is, too much subjective influence. When AHP is used to determine the weight, it is usually based on the experts' opinions to score the impact degree of the index value. Although this scoring mechanism is a certain objective and realistic subjective judgment for the evaluation, it has human subjective factors and is relatively suitable for qualitative indexes. For quantitative indexes, CV is another statistic that can objectively reflect the change of indicator value and measure the variation degree of each observation value in the data^27,29^. It can be used to calculate the influence degree of each factor and the objective weight. From the calculation results, it can be found that the fault distribution and water pressure gain a high weight in AHP, because from the geological point of view. On the one hand, the tectonic action controls the development of structural fractures and affects the water permeability of rocks; on the other hand, it controls the collection and main channel of groundwater. Therefore, the distribution of faults plays an important role in the study of water abundance. Moreover, water pressure is an important evaluation index in a confined aquifer, which can reflect the water yield of the aquifer. However, from the calculation results of CV, the weight of these two factors has become lower due to the quantitative indexes of these factors in the study area have little change. While the fault scale index has improved its weight in CV due to large data differences. In order to better reflect such objective facts, therefore, a more reasonable weight can be obtained by using the combination of AHP–CV.

**Figure 6:**
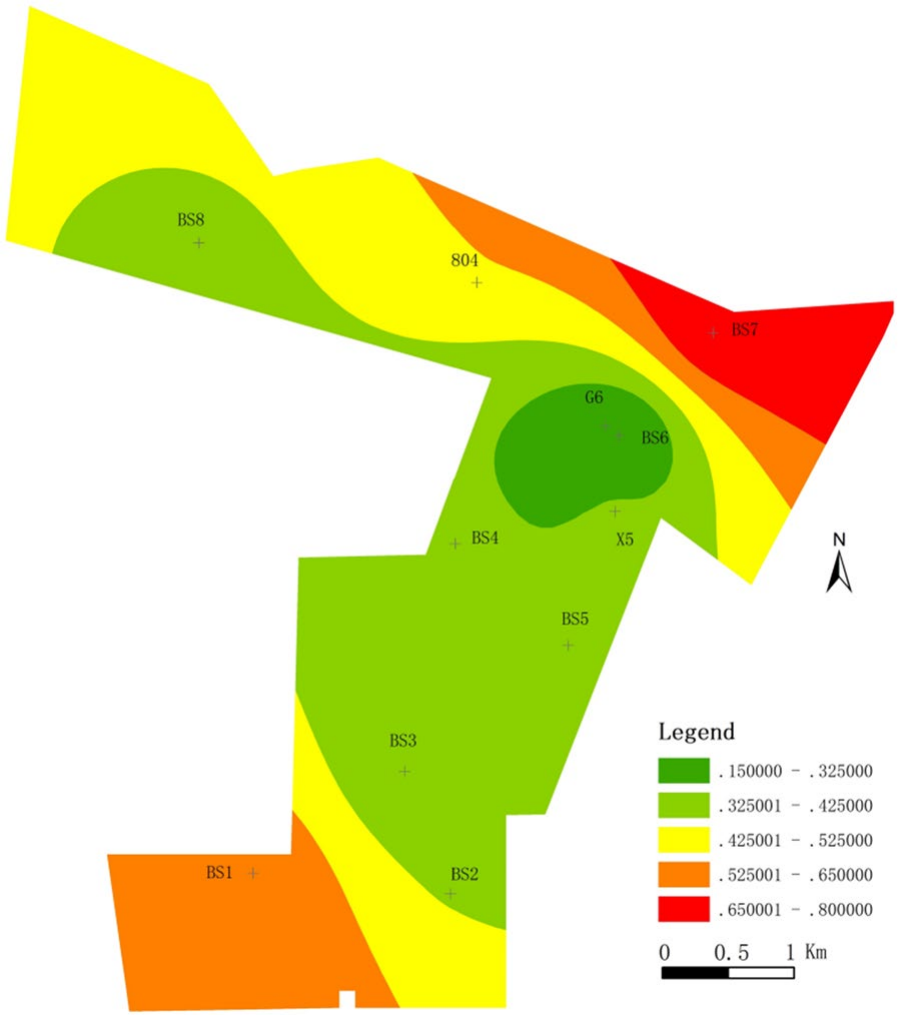
Specific yield map of the study area.

In contrast to other research, we can use specific yield as a verification^31,32^. The specific yield is usually used to evaluate the water-yield of a regional aquifer, but it is only for a homogeneous aquifer. For the karst aquifer, karst development is not uniform, which leads to a great difference in the water-yield of the aquifer^33,34^. In the mining area, because the WYP of karst aquifer is particularly inhomogeneous, the application of specific yield to the evaluation of water-yield of karst aquifer needs further discussion. Generally, on the premise of considering many geological factors, the comprehensive evaluation of the water-yield of karst aquifer can meet the actual needs. Therefore, in this paper, we select multiple factors to evaluate the WYP and still use the specific yield as the basis for verification. Through the comparison of results (Fig. 6), they have similar distribution laws, and the zoning results of WYP are much more detailed. However, the specific yield of boreholes BS1 and BS2 is not consistent with the results of the WYP, and the accuracy of the evaluation results is 80%.

For karst aquifer, the water content of aquifer is closely related to karst development^35,36^. Therefore, geological structure and aquifer are taken into account in the selection of evaluation indexes. The geological structure controls the development of karst to a certain extent, and the karst fissures and karst fractures in the karst development area will also be relatively developed, forming a good water storage space and migration space. When there are supply and discharge conditions, karst development is stronger. At the same time, it shows that the region has better hydrological cycle conditions. On this issue, we found that the hydrochemical characteristics of karst aquifer in the study area are similar to the results of WYP model, which reflects the close relationship between the karst development degree and the evaluation model.

**Figure 7:**
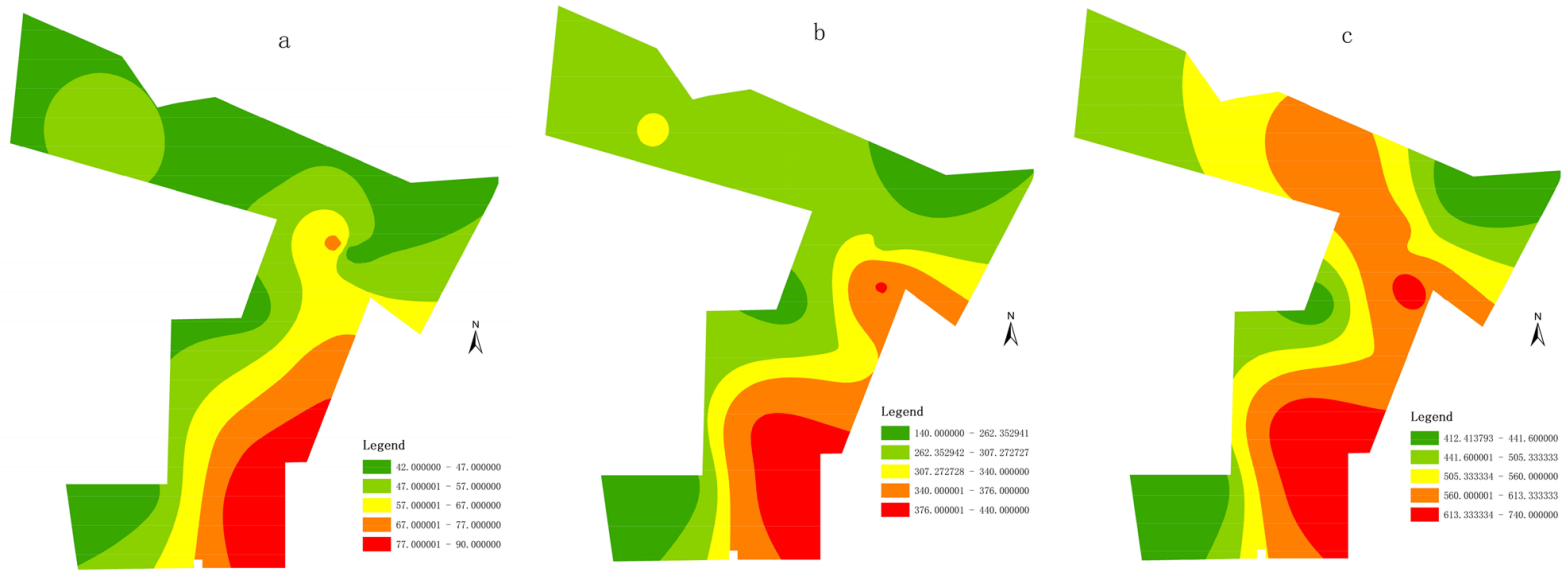
The zone map of concentration distribution: (**a**) Ca^2+^, (**b**) HCO_3_^−^ and (**c**) TDS.

According to the concentration distribution of Ca^2+^, HCO_3_^−^ and TDS, they are higher in the southeast of the study area, and gradually lower in the north (Fig. 7). The energy input of soluble rock, water, and CO_2_ system is realized by the continuous infiltration of groundwater. The material output of this system, namely, the discharge of CaCO_3_, must also depend on the runoff and discharge of water^37–39^. Therefore, the circulation alternation of groundwater is necessary and enough condition to ensure karst development. The overall topography of the mining area is high in the South and low in the north. The groundwater flow direction of Ordovician limestone karst aquifer is also from south to north. For the same karst aquifer, under the same supply, runoff and discharge conditions, such differences reflect the uneven development of karst. However, the concentration of Ca^2+^ and HCO_3_^−^ in the south of the study area is higher than that in the north, indicating that the groundwater circulation alternation in the north is better than that in the south. For one thing, under the same supply conditions, especially the atmospheric rainfall supply, the intensity of infiltration supply in the north is greater than that in the South; For another thing, after infiltration supply, the mobility of groundwater is also better than that in the south. Based on the geological structure of the study area, we can also find that there are many faults and structures in the north of the mining area (Fig. 1), so that the infiltration supply conditions in the north of the study area are better than those in the south, and there are more underground water diversion channels. Thus, the karst development in the north of the study area is stronger than that in the south. The karst fractures and karst fissures are developed and the water-yield is stronger.

Generally, there are fewer Ordovician boreholes in the mine area. On the one hand, because the upper group coal is mainly mined in the early stage of mining, the threat of water inrush from the coal mine is mainly concentrated in the roof water hazard and goaf water. However, with the mining of the lower group coal, the distance between the coal seam and the Ordovician limestone aquifer becomes shorter, and the mining is threatened by water inrush from the Ordovician limestone aquifer. However, the number of boreholes in a mine is limited in relation to the exposure of Ordovician limestone. Therefore, there is an insufficient understanding of the WYP of Ordovician limestone aquifer. At the same time, the development of karst is also difficult to use a single index as the evaluation of WYP. In summary, it is feasible to use the water yield index method to evaluate the water yield of a karst aquifer. First, the combination of AHP and CV can reduce the influence of subjective judgment. Second, the selected evaluation indexes can comprehensively reflect the nature of karst aquifers. Third, the development degree of karst affects the evaluation effect of the model. The specific yield as a reference basis only has a small reference value, but it can be used as the effect verify of model evaluation in a certain area.

## Conclusions

This paper presents an improvement model for evaluating WYP of the karst aquifer. Based on the analysis of the hydrochemistry composition of karst groundwater, it can be found that the WYP law of karst aquifer is related to the degree of karst development. Therefore, it is very important to select indicators that can reflect karst development in the evaluation system. In addition, reasonable weight calculation is also necessary. In order to eliminate the influence of subjective factors, the objective weight can effectively adjust the evaluation results of the model. The WYP area was divided into five grades by natural discontinuity classification, which are strong water-yield area, relatively strong water-yield area, medium water-yield area, relatively weak water-yield area, and weak water-yield area. through the proposed evaluation model of water-yield, we can make a reasonable evaluation of the water-yield law of karst aquifer, which has an extremely important theoretical guiding significance and practical value for mine safety production. In addition, the model is a feasible method for groundwater exploration in a karst area.
